# Surveying Moths Using Light Traps: Effects of Weather and Time of Year

**DOI:** 10.1371/journal.pone.0092453

**Published:** 2014-03-17

**Authors:** Dennis Jonason, Markus Franzén, Thomas Ranius

**Affiliations:** 1 IFM Biology, Division of Ecology, Linköping University, Linköping, Sweden; 2 Swedish University of Agricultural Sciences, Department of Ecology, Uppsala, Sweden; 3 UFZ Helmholtz Centre for Environmental Research, Department of Community Ecology, Halle, Germany; University of Regina, Canada

## Abstract

Light trapping is an ideal method for surveying nocturnal moths, but in the absence of standardised survey methods effects of confounding factors may impede interpretation of the acquired data. We explored the influence of weather, time of year, and light source on nightly catches of macro moths in light traps, and compared four strategies for sampling by estimating observed species richness using rarefaction. We operated two traps with different light sources for 225 consecutive nights from mid-March to the end of October in eastern Germany in 2011. In total, 49 472 individuals of 372 species were recorded. Species richness and abundance per night were mainly influenced by night temperature, humidity and lamp type. With a limited sample size (<10 nights) it was slightly better to concentrate sampling on the warmest summer nights, but with more sampling nights it was slightly better to sample during the warmest nights in each month (March to October). By exploiting the higher moth activity during warm nights and an understanding of the species' phenology, it is possible to increase the number of species caught and reduce effects of confounding abiotic factors.

## Introduction

Insects are the most species-rich taxon with about one million species described worldwide, corresponding to more than half of all known species [1], [2]. Due to their high ecological diversification and short generation times, insects are useful indicators of environmental change [3], [4]. Lepidoptera (butterflies and moths) is one of the largest insect orders with 160,000 described species, of which 95% are moths [5], [6]. Moths play important roles in many ecosystems as pollinators, herbivores, and prey for a wide range of species such as birds and bats [7], [8]. The distribution and ecology of moths are well known in comparison to many other invertebrates [9]. In recent decades, steep declines of moth populations have been observed. For instance, in Great Britain, the abundance of macro-moths decreased by 28% between 1968 and 2007 [9] and similar negative trends have been found in Sweden [10] and the Netherlands [11]. Such declines are expected to have cascading effects at both higher (bats, birds) and lower (plants) trophic levels due to the keystone role of moths in many ecosystems [8], [12].

The most widely applied method to survey moths is to use light traps, which exploit their attraction to artificial light [10], [13]–[16]. Light traps can be designed in various ways and operated using different light sources; both of these modifications are known to affect trap performance [17], [18]. Weather factors, such as temperature, and rainfall, also influence catch size [19], [20]. However, there have been few rigorous evaluations of trap efficiency (e.g. [20]–[22]). This is problematic because methodological inconsistencies may restrict the value of monitoring data by complicating attempts to assess trends and compare results across studies and regions [23].

In the presented study we investigated efficient, standardised ways of surveying macro-moths using light traps in an agricultural landscape in Germany. We determined effects of four weather parameters (temperature, wind speed, air humidity, and precipitation), lamp type, and time of year on the species richness and numbers of moths trapped per night. Based on the outcome of these analyses, we formulated different strategies for sampling at a specific temperature or time of year, and estimated the number of moth species caught using these strategies.

## Materials and Methods

All legal permits required to collect field data were authorised by the municipality of Döbeln (Unteren Naturschutzbehörde). The landowner was informed about the study, and approved it in advance. The study did not involve catching any protected species.

The experiment was established near the village Auterwitz (N 51‘09’37.40; E 18‘12’00.17) in the German federal state of Saxony. The area is dominated by intensive agriculture (>90% arable land) that is typical for large regions of central and northern Europe. We used two Ryrholm light traps [24] with different light sources: a 250 W mercury lamp (Sylvania HSL-GW) and a 40 W ultra-violet fluorescent tube (Wemlite TT40WX). The lights attract the moths to the traps where they fall down through a funnel and into a box where they rest until identification. To avoid damaging the collected specimens, the box contained egg trays between which the moths could hide. The traps were placed in an open grassland area at a distance of 40 m to one another. The attraction of light traps decreases with distance, and is low at distances exceeding 20 m [25]–[27], and the 40 m distance was chosen to minimise trap interference while securing similar environmental conditions near the traps. To avoid possible influence of the local site on trap performance, the traps were rotated between the two positions three times within the sampling period.

We operated the traps every night from 21 March to 31 October, 2011, for a total of 225 sampling nights for each lamp type. The lamps were automatically switched on at dawn and off at dusk using twilight sensors. The traps were emptied every morning. All macro-moths (predominantly belonging to the families Noctuidae and Geometridae) were identified to species level using several identification guides [28]–[33]. To test the influence of weather on trap performance, we used 16 parameters reflecting weather conditions for each trapping night, measured at a climate station in Salbitz, approximately 10 km south of the sampling location. Parameters were calculated as averages of one measure per hour from 10 pm to 4 am. From these 16 parameters, we used pairwise Pearson's correlations to select four independent (r<0.40, i.e. weak correlation [34]) ones for further analyses: average temperature, average wind speed, average air humidity and total precipitation, all of which are known to affect light trapping of moths (e.g. [20], [28]). Moonlight is also known to affect light trap efficiency [20], [36], and was included as a factor in the correlation matrix. However, due to its correlation with temperature and because recommendations for sampling are easier to make using temperature, moonlight was excluded from further analyses.

### Statistical analyses

All statistical analyses were conducted in R v. 3.0.0 [37]. We analysed the relationships of two catch parameters (the number of moths and moth species caught per night: abundance and species richness, respectively) with temperature, wind speed, air humidity, precipitation, and the two light sources (the 250 W mercury lamp and 40 W ultra-violet fluorescent tube), using generalised linear mixed models (GLMM) with a Poisson error distribution implemented in the lme4 package [38]. Date and trap position were included as random factors. No interactions were considered in these models. Models were compared using an information theory approach based on Akaike's information criterion (AIC). We used AIC_c_ (i.e. AIC with a second-order correction for sample size) and Akaike weights (i.e. the probability of the best fit among the set of candidate models), derived using the MuMIn package [39], to assess the relative fit of models with different combinations of parameters to the data. Model averaging was performed on all models with ΔAIC_c_<8 to circumvent the problem of model selection uncertainty, in which the contribution of each candidate model to the average parameter estimates is proportional to the model weight. Before model averaging, the global model was standardised to a mean of 0 and an SD of 0.5 using the arm package [40]. Standardisation enables the interpretation of parameter estimates in relation to each other by placing them on a comparable scale, irrespective of whether the parameters are categorical or continuous [41].

To identify the periods of the year most important to cover for trapping the most species of moths, we analysed changes in community between consecutive months. As a measure of monthly species turnover, two types of incidence based (dis)similarity indices were calculated: the Jaccard index and a modified version of the Simpson index (*sensu* [42]). Both indices range from 0 to 1, where 1 equals 100% similarity. The Jaccard index is calculated as:
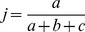
(1)where *a* is the number of shared species between two consecutive months, and *b* and *c* are the number of unique species recorded in each month (i.e. the number of shared species divided by total species richness). The modified Simpson index is calculated by dividing the number of shared species by the number of species recorded in the month with the lowest number of species (*d*):



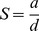
(2)Thus, this index distinguishes changes in species richness from changes in species turnover [43]. For example, the total species richness for April and May was 178, of which 32 species were unique for April, 119 were unique for May and 27 species were shared between months. The resulting Jaccard index is 0.15, indicating low compositional similarity. However, the modified Simpson index is 0.46, which is much higher since the large difference in the number of species does not affect the outcome.

We compared estimates of observed species richness obtained from four sampling strategies using rarefaction. We tested strategies in relation to night temperature and time of year, because species composition varied over the season and temperature was the most important weather factor affecting species richness. The activity of most species peaked during the summer, suggesting that sampling concentrated in this time period would be most efficient. The strategies were: i) ALL – sampling in all nights (n = 225), ii) SUMMER – sampling only in nights in June, July and August (n = 92), iii) WARMEST SUMMER – sampling only in the warmest nights in June, July and August (n = 23, temperature >16.4°C), and iv) WARMEST MONTHLY – sampling only in the warmest nights of each months (n = 56). The warmest nights belonged to the warmest quartile of each time period.

## Results

**Figure 1:**
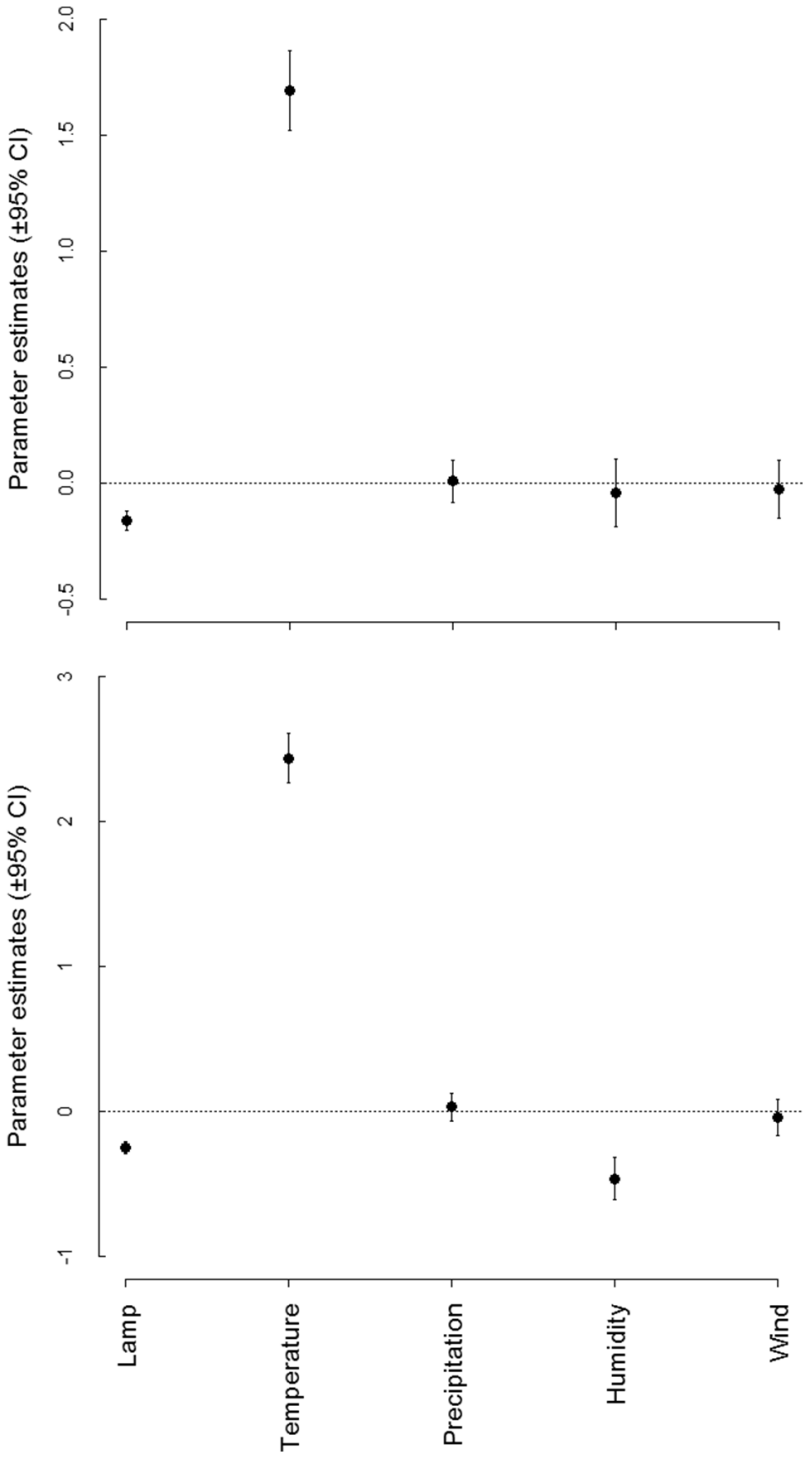
Effects of lamp type and weather parameters on moth species richness (a) and abundance (b). The parameter estimates and 95% CI are derived from model averaging. All estimates are on a comparable scale due to standardisation of the models.

In total, we caught 49472 individuals belonging to 372 species. Most species belonged to two families: Noctuidae (46%) and Geometridae (33%). The two most abundant species were the two noctuids *Xestia c-nigrum* and *Hoplodrina octogenarian*, comprising 14% and 10% of all individuals caught, respectively. Most species were rare; singletons of about 20% of all species, and five or less individuals of about 40% of the species, were recorded (Table S1). Species richness and abundance per night were positively correlated with temperature, and abundance was negatively correlated with air humidity (Figure 1; Table 1). There were also significant differences in catches between the two lamps used; the trap with the 250 W mercury lamp caught 29953 individuals representing 334 species, while the trap with the 40 W ultra-violet fluorescent tube caught 19519 individuals representing 299 species. The standardised estimate of temperature effects was an order of magnitude larger than the standardised estimate of lamp type effects, suggesting that temperature is the most important factor for trapping moths. Species richness and abundance were not significantly influenced by rain or wind speed.

**Table 1:** Standardised parameter estimates for the models of the effects of lamp type and climate on trap performance including standard error (SE), 95% confidence interval (CI) and relative variable importance for moth species richness and abundance.

			95% CI		Relative variable
	Estimate	SE	Lower	Upper	importance
Species richness					
Lamp type*	−0.159	0.022	−0.159	−0.159	1.00
Temperature (°C)	1.693	0.088	1.683	1.703	1.00
Air humidity (%)	−0.040	0.073	−0.138	0.057	0.41
Wind (m/s)	−0.026	0.064	−0.102	0.050	0.34
Precipitation (mm)	0.010	0.047	−0.024	0.044	0.28
Intercept	2.624	0.106	2.625	2.627	
Abundance					
Lamp type*	−0.248	0.010	−0.248	−0.248	1.00
Temperature (°C)	2.440	0.147	2.419	2.460	1.00
Air humidity (%)	−0.460	0.154	−0.560	0.360	0.99
Wind (m/s)	−0.038	0.104	−0.148	0.072	0.32
Precipitation (mm)	0.032	0.095	−0.062	0.126	0.31
Intercept	3.819	0.186	3.817	3.820	

* A negative estimate indicates catches with lower species richness or abundance in the trap with the 40 W ultra-violet black light bulb than in that with the 250 W mercury bulb.

**Figure 2:**
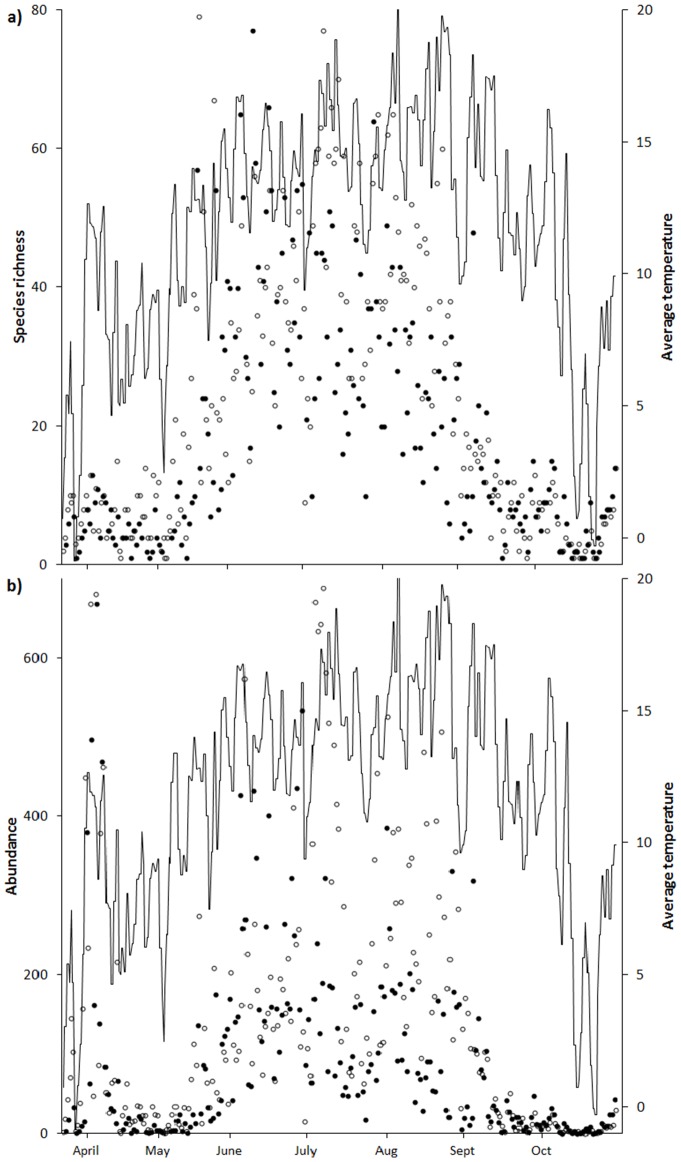
Nightly catches of moth species (a) and individuals (b). Different symbols represent different light sources (○ = 250W; • = 40W) and the line represents temperature.

**Figure 3:**
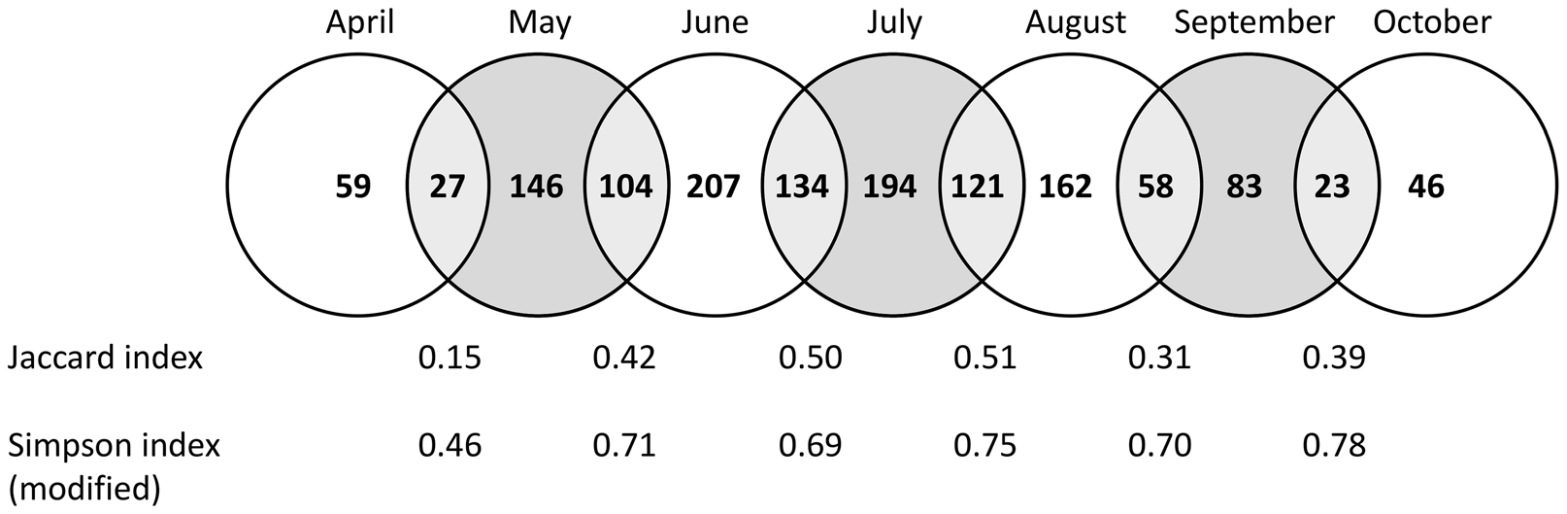
The number of species caught in each month with each light source, and the number of shared species between consecutive months. The Jaccard and Simpson indices reflect similarities in species composition and species richness, respectively. Total species richness for the period was 372 species.

**Figure 4:**
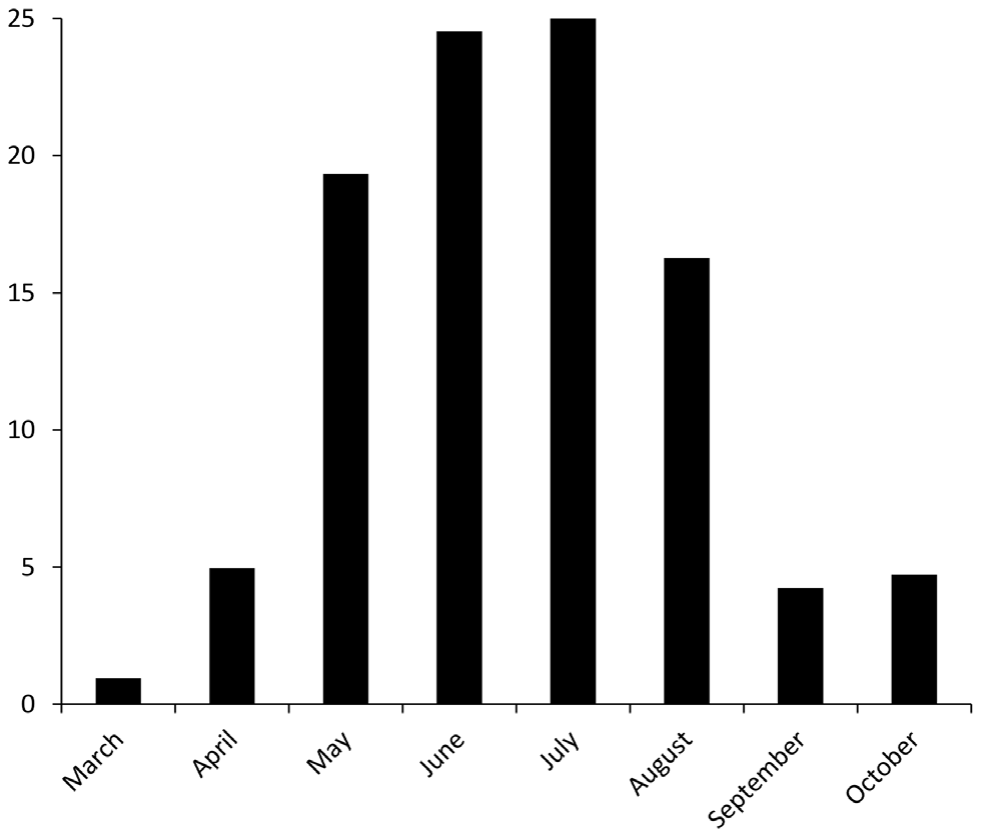
Proportion of species with a peak in abundance in each month.

The number of species captured per night increased dramatically in mid-May and remained high (∼40 species) throughout August (Figure 2a). Catches of about 25% of species peaked during spring (March – May), 65% during summer (June – August), and about 10% during autumn (September – October) (Figure 3). Moth abundance displayed a similar pattern to species richness, but showed a clear peak in the beginning of April (Figure 2b). The similarity indices showed that there were large changes in species composition over time; the Jaccard index between two consecutive months ranged from 15 to 51% similarity and the modified Simpson index ranged from 46 to 78% similarity (Figure 4).

**Figure 5:**
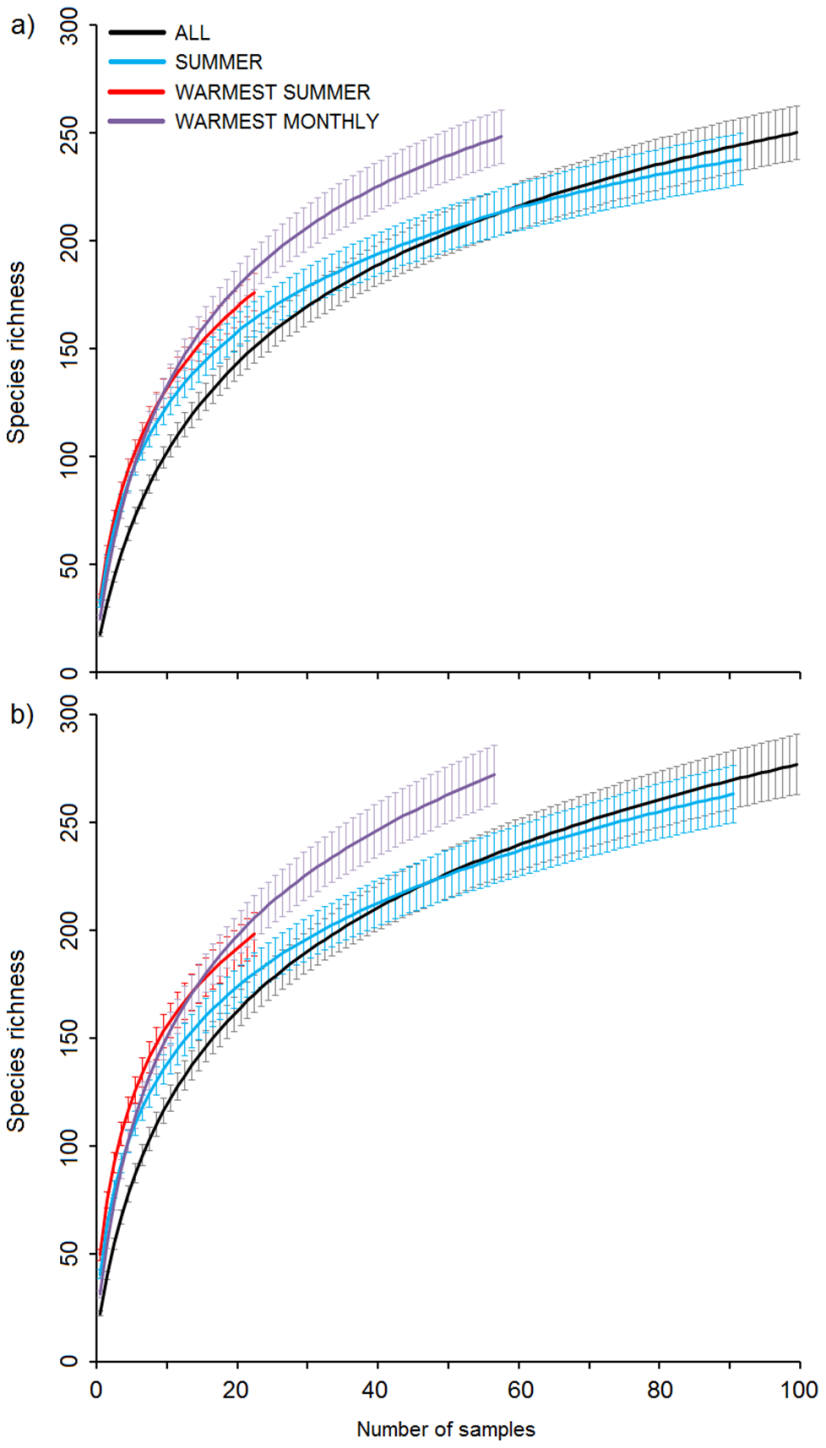
Sample-based rarefaction curves with 95% confidence intervals generated from data obtained using four sampling strategies and the traps with a 40 W black light bulb (a) and a 250 W mercury lamp (b). The strategies were: ALL – sampling during all nights (n = 225); SUMMER – sampling only in nights in June, July and August (n = 92); WARMEST SUMMER – sampling only in the warmest nights in June, July and August (n = 23); and WARMEST MONTHLY – sampling only in the warmest nights in each month (n = 56). Warm nights belonged to the warmest quartile of each time period. The figure is restricted to a maximum of 100 sample nights.

More species were captured when sampling on warm nights than when sampling, during the same number of nights, with no specific temperature restrictions (Figure 5). At low sample sizes (<10 nights), slightly more species were captured when sampling was restricted to the warmest summer nights, but at larger sample sizes (>10 nights), slightly more species were captured when sampling during the warmest nights of each month. With the latter, ‘WARMEST MONTHLY’ strategy, after 30 nights we captured 60% of all species when using the trap with the 250 W mercury lamp, and 55% when using the trap with the 40 W ultra-violet fluorescent tube.

## Discussion

By analysing moth trap catches in relation to light source, weather parameters and time of the year, our main findings are that (1) observed species richness and abundance were affected by the temperature and light source, (2) moth abundance decreased with increases in air humidity, and (3) most moth species were most active during the summer. With a low sample size (<10 nights), slightly more species were captured when sampling was limited to a few warm summer nights, but with larger sample sizes, slightly more species were captured when sampling was limited to the warmest nights in each month (from March to October).

That the highest numbers of moths were captured during warm nights is consistent with previous studies [20], [36] and may be attributed to the positive correlation between the activity of ectothermic species and ambient temperature [44], [45]. The finding that air humidity was negatively correlated with moth abundance conflicts with a previous report of positive correlations between air humidity and numbers of both individuals and species captured [35]. This discrepancy could be due to high humidity at night often resulting in fog in our study area, which may limit trap efficiency by reducing light from the traps and/or decrease moth activity.

The trap with the mercury lamp was considerably more effective in catching moths than the trap with the ultra-violet fluorescent tube, in accordance with findings by Bates et al. [21]. However, the pattern conflicts with reports that more moths are generally attracted to artificial light with relatively short wavelengths [35], [46] as the fluorescent tube emitted light with shorter wavelengths (300–460 nm) than the mercury lamp (400–600 nm). The likeliest explanation for the difference in trapping efficiency is the higher power of the mercury lamp (250 W vs. 40 W), and hence greater contrast it provided between light from the trap and background illumination [20].

Species richness per night was generally higher from mid-May to the end of August. This may be because higher temperature increases flight activity and the numbers present in an area of both species and individuals [44], [45]. In September, both species richness and abundance were lower than in summer nights with similar temperatures. Thus, the higher species richness in summer is not only due to temperature *per se*, but can be at least partly explained by the presence of more species in the area during summer. Phenological variation in species composition was high between months, especially from April to May. One reason for this was that it was a transient peak of nearly 6000 individuals of the genus *Orthosia*, (*ca.* 12% of all recorded individuals) during the first week of April. This peak was likely synchronized with the blooming of willow (*Salix* spp.), the major floral resource in the spring and one of the genus' host plants [47], as timing of adult emergence is an adaptive trait that depends on nectar availability and host plant resources [48], [49]. Thus, phenological factors, including adaptations to nectar availability, seem to have a strong effect on patterns of moth species richness and abundance.

Sampling during the warmest nights was more efficient than sampling during the same number of randomly selected nights without regards to temperature, because random sampling also includes nights with poor conditions and therefore lower trap catches. With a limited sample size it was slightly better to concentrate sampling on the warmest nights of the summer, but with larger sample sizes it was slightly more efficient to sample over the entire season during the warmest nights of each month. With a small sample size it is advantageous to take samples when species richness is at its maximum, whereas larger sample sizes can also include species that are only active in the spring or autumn.

Sampling approaches used in ecological studies on moths vary greatly. Sometimes samples are collected at regular intervals (once per month or per week) [25], [50], [51], sometimes only on warm nights [15], [16], [50], and sometimes irrespective of temperature or temperature is not mentioned in reports [52]–[54]. The latter strategy is problematic, given the strong effect of temperature found in the present study. Furthermore, in studies where sampling has been performed only above a predefined temperature limit, the limit has remained constant over the season [12], [51], [55], [56]. Often this means that sampling is concentrated in the summer, which results in a sampling strategy similar to one of the two optimum strategies that we identified.

Surveys of species richness and abundance are vital for assessing the current status and trends of moth communities. For butterflies in temperate regions that fly during the day, there are strict criteria for the temperatures at which monitoring should be performed [57], but no such criteria have been established for moths. Our results suggest that temperature is the most important factor to consider in moth trapping using light traps, and that temperature requirements for sampling should be adapted to the time of year.

## Supporting Information

Table S1 (XLSX)
